# Editorial Focus: CFTR‐dependent bicarbonate secretion by Calu‐3 cells

**DOI:** 10.14814/phy2.13691

**Published:** 2018-05-15

**Authors:** Ronald C. Rubenstein

**Affiliations:** ^1^ Division of Pulmonary Medicine and Cystic Fibrosis Center The Children's Hospital of Philadelphia Department of Pediatrics Perelman School of Medicine at the University of Pennsylvania Philadelphia Pennsylvania

## Abstract

Cystic Fibrosis is an autosomal recessive multisystem disease caused by the severely impaired or absent function of the Cystic Fibrosis Transmembrane Conductance Regulator, CFTR. While CFTR is best recognized as a Cl‐ channel, impairment of CFTR‐dependent bicarbonate transport is increasingly recognized as a potentially key element in the pathophysiology of airways disease in CF. It is in this context that the discussed in this Editorial Focus by Huang, Kim, et al. has significant relevance to CF and our understanding of the pathophysiology of CF airway.

Cystic fibrosis is an autosomal recessive multisystem disease caused by the severely impaired or absent function of the Cystic Fibrosis Transmembrane Conductance Regulator, CFTR. While CFTR is best recognized as a Cl^−^ channel that regulates salt and water transport at the apical surface of epithelia, and this Cl^−^ transport function is often used to determine whether mutations of CFTR are disease causing (Johns Hopkins CFTR mutation database, http://www.cftr2.org), it is also recognized that CFTR can transport or regulate the transport of other anions, including bicarbonate. In fact, while controversial, some data suggest that CFTR mutations that are selectively deficient in bicarbonate transport or the regulation of bicarbonate transport by partner channels may be associated with a more severe CF phenotype including pancreatic insufficiency (Choi et al. 2001; Ko et al. 2002).

Impairment of CFTR‐dependent bicarbonate transport is increasingly recognized as a potentially key element in the pathophysiology of airways disease in CF. The airway surface liquid of the CF airway has a lower pH than that of non‐CF airways (Pezzulo et al. 2012), which may result in part from decreased bicarbonate secretion onto the airway surface when CFTR is absent. Similarly, the lack of bicarbonate may inhibit innate bacterial killing (Pezzulo et al. 2012) and, in that way, further contribute to the chronic bacterial colonization that results from the CF airway's impaired mucociliary clearance.

The importance of defective CFTR in causing abnormal mucus secretion from the airway submucosal glands is also increasingly acknowledged. This was initially recognized in studies demonstrating that inhibition of both bicarbonate and Cl^−^ secretion by submucosal glands led to impaired mucociliary clearance (Ballard et al. 2002), and that the submucosal glands in CF airway explants were deficient in adrenergic‐stimulated mucus secretion (Joo et al. 2006). More recent data examining the larger airways from CF pigs studied ex vivo show that mucociliary transport is compromised by strands of mucus that fail to readily detach from the gland. Interestingly this phenomenon is also seen in the larger airways of non‐CF control pigs when studied under bicarbonate‐free conditions where glandular liquid secretion has also been inhibited (Hoegger et al. 2014). The potential primacy of bicarbonate in the appropriate secretion (Yang et al. 2013) and structural and transport (Chen et al. 2010) properties of secreted mucus has also been suggested.

It is in this context that the work by Huang et al. (2018) has significant relevance to CF and our understanding of the pathophysiology of CF airway. As a reasonable model of airway glandular serous/fluid secreting cells, these investigators studied Calu‐3 cells, a non–small cell lung adenocarcinoma cell line that forms polarized epithelial monolayers and expresses a number of markers of submucosal glands. Calu‐3 cells have two potential mechanisms by which bicarbonate secretion onto the apical/glandular luminal surface may occur. In one mechanism, bicarbonate may be directly secreted onto the apical surface through CFTR that is present in the apical membrane. Alternatively, CFTR may predominantly transport Cl^−^ onto the apical surface, and the Cl^−^ is subsequently exchanged for bicarbonate by Pendrin (SLC26A4); analogous mechanisms of Cl^−^/bicarbonate exchange mediated by SLC26 family members and involving CFTR are operative in the exocrine pancreas (Ko et al. 2002).

In rigorously performed and controlled studies, the investigators demonstrated that the depletion of Pendrin expression did not alter basal or cAMP (forskolin) stimulated secretion of fluid or bicarbonate, nor did it alter cAMP‐stimulated short circuit current (Isc) or bicarbonate flux. Importantly, they demonstrated that the depletion of Pendrin did inhibit Cl^−^/bicarbonate exchange at the apical membrane. Similarly, the depletion of CFTR expression or pharmacologic inhibition of CFTR decreased much of the apparent bicarbonate transport. Together, these data support the hypothesis that CFTR is the predominant transporter for bicarbonate at the apical surface of the serous cell‐like Calu‐3 cells. As these data are apparently discrepant from the results of previous work suggesting that the depletion of Pendrin rather than CFTR decreased the pH of Calu‐3 cell secretions (Garnett et al. 2011), the investigators attempted to address these discrepancies but found no obvious experimental explanation other than the possibility that the depletion of CFTR function was less complete in the previous work (~28% residual CFTR function, (Garnett et al. 2011)) versus the present work (~5% residual CFTR function).

From a potential therapeutic standpoint, the present data clearly suggest that correcting the aforementioned mucus secretory defect(s) in CF may require targeting the dysfunction of CFTR in the submucosal glands. In contrast, these data do not exclude the viability of Pendrin as a target of pharmacotherapy in CF. In primary human airway epithelial cells, pendrin expression and Cl^−^/bicarbonate exchange function is induced by inflammation (IL‐17A, (Adams et al. 2014); IL‐13 (Haggie et al. 2016)). Interestingly, and perhaps counterintuitively, it was inhibition of Pendrin function that increased the depth of the airway surface liquid in these experiments. Such increases in airway surface liquid by inhibition of Pendrin, would be predicted to improve defective mucociliary clearance in CF as do osmotic agents of clinical benefit such as inhalation of hypertonic saline. Importantly these mechanisms of improving airway clearance would be agnostic to the underlying CFTR mutation, and therefore potentially beneficial to those with CF caused by mutations that are not amenable to CFTR modulator therapies.

## Conflict of Interest

The author has no conflicts of interest relevant to this work.
